# BC Walks: Replication of a Communitywide Physical Activity Campaign.

**Published:** 2006-06-15

**Authors:** Bill Reger-Nash, Patricia Fell, Deborah Spicer, Brian D Fisher, Linda Cooper, Tien Chey, Adrian Bauman

**Affiliations:** Department of Community Medicine; Community Health Services, United Health Services Hospitals, Johnson City, NY; Healthy Heart Program, New York State Department of Health, Albany, NY; New York State Department of Health/Epidemiology Department, State University of New York (SUNY), University of Albany, Albany, NY; Media-Based Communication Projects, Department of Community Medicine, West Virginia University, Morgantown, WVa; Centre for Physical Activity and Health, School of Public Health, University of Sydney, Sydney, Australia; Centre for Physical Activity and Health, School of Public Health, University of Sydney, Sydney, Australia

## Abstract

**Introduction:**

Individuals not engaging in recommended amounts of moderate-intensity physical activity are deemed insufficiently active and are at greater risk of chronic disease. Social marketing strategies may promote positive changes in physical activity levels among insufficiently active individuals.

**Methods:**

A quasi-experimental design was used to determine whether the results of a previous communitywide physical activity social marketing campaign conducted in Wheeling, WVa (population, 31,420) could be replicated in the larger community of Broome County, New York (population, 200,536). BC Walks promoted 30 minutes or more of moderate-intensity daily walking among insufficiently active residents of Broome County, New York, aged 40 to 65 years. Promotion activities included paid advertising, media relations, and community health activities. Impact was determined by preintervention and postintervention random-digit–dial cohort telephone surveys in intervention and comparison counties. We assessed demographics, walking behavior, moderate and vigorous physical activity, and campaign awareness.

**Results:**

The paid advertising included 4835 television and 3245 radio gross rating points and 10 quarter-page newspaper advertisements. News media relations resulted in 28 television news stories, 5 radio stories, 10 newspaper stories, and 125 television news promotions. Exposure to the campaign was reported by 78% of Broome County survey respondents. Sixteen percent of Broome County participants changed from nonactive to active walkers; 11% changed from nonactive to active walkers in the comparison county (adjusted odds ratio, 1.71; 95% confidence interval, 0.99–2.95). Forty-seven percent of Broome County respondents reported any increase in total weekly walking time, compared with 36% for the comparison county (adjusted odds ratio, 1.66; 95% confidence interval, 1.14–2.44).

**Conclusion:**

The BC Walks campaign replicated the earlier Wheeling Walks initiative, although increases in walking were smaller in the BC Walks campaign.

## Introduction

Physical activity levels in the United States have remained relatively unchanged during the past 20 years (1). Physical inactivity is responsible for approximately 250,000 deaths annually in the United States (2) and is likely a major contributing factor in the epidemics of overweight, obesity, and diabetes (3). In 1995 and 1996, major public health reviews recognized the health benefits of moderate-intensity physical activity, such as walking 30 minutes daily (4-5). The recommendation of the Centers for Disease Control and Prevention (CDC), American College of Sports Medicine (ACSM), and the U.S. surgeon general is to engage in moderate-intensity physical activity at least 30 minutes per day 5 days per week. Individuals not meeting the recommendation are deemed insufficiently active and at greater risk for chronic disease (4-5).

The pervasiveness of physical inactivity represents a populationwide problem (6). Most health promotion efforts, focused on individual and small group programs, have little impact (7). By contrast, populationwide methods to increase moderate-intensity daily physical activity represent a primary prevention strategy to address the growing problem of energy imbalance as well as the increases in chronic disease morbidity that confront industrialized societies (5). Early populationwide cardiovascular interventions had limited capacity to promote physical activity (8-11). More recent campaigns, including CDC's national VERB campaign, have documented significant changes in physical activity (12-18).

Social marketing strategies may promote populationwide public health change (19). Through the use of targeted mass media and environmental supports, social marketing strategies can increase public awareness and set a public agenda for improving healthy lifestyles (19-22).

One social marketing initiative, Wheeling Walks, involved paid mass media, public relations, and community health activities, along with efforts focused on policy and environmental changes in the community of Wheeling, WVa (23-25). This model campaign targeted older adults with 8 weeks of media messages encouraging 30 minutes or more of daily moderate-intensity walking. Ninety percent of individuals surveyed in Wheeling reported exposure to the campaign through mass media, and the campaign resulted in a 14% increase in the number of participants who changed from nonactive to active walkers.

Although model programs may succeed in their own communities, they frequently fail in other communities. To determine the effectiveness of the Wheeling Walks campaign methodology in another community, BC Walks was implemented in Broome County, New York. This paper reports on the short-term evaluation of the BC Walks campaign.

## Methods

### Target population

BC Walks was conducted in Broome County, New York (population, 200,536). The campaign targeted insufficiently active adults aged 40 to 65 years, who comprise 32% of the county's population, or 36,080 adults. Chautauqua County, New York (215 miles from Broome County), was selected as the comparison community because it has similar demographic characteristics and physical activity levels (Table 1), and it has separate and distinct newspaper, radio, and network and cable television markets. The Institutional Research Board for the Protection of Human Subjects of United Health Services Hospitals approved the intervention.

### Design

BC Walks used a quasi-experimental design and social marketing principles to promote walking during the 8-week period of May 1 through June 26, 2003. The campaign consisted of paid media, public relations, including a speakers' bureau, and community health activities.

We purchased 953 30-second advertisements on prime-time network television (equal to 4835 gross rating points) and 1645 60-second radio advertisements (3245 gross rating points). (Gross rating points are a media industry measure to assess message penetration.) In addition, we purchased 10 quarter-page advertisements in the local daily newspaper and 1314 30-second advertisements on cable television.             

Public relations focused on communicating the campaign message in news media. Community health activities were designed to provide social networks, to offer social support, and to reinforce the campaign message.

### Costs

Total intervention expenditures were $155,656 (without evaluation). Media costs were $126,676, with $70,895 spent on network television advertisements, $13,900 on cable television advertisements, $32,186 on radio advertisements, and $9675 on newspaper advertisements. Personnel costs were $29,000. Thus, $4.31 was expended for each of the 36,080 targeted residents of Broome County.

### Measurement

The impact of the intervention was determined by baseline and follow-up random-digit–dial telephone surveys in Broome and Chautauqua counties. The surveys were designed specifically to evaluate BC Walks. One month before the campaign (baseline), we screened the first respondent aged 40 to 65 years to answer the telephone at a household to determine the person's physical activity level. Respondents who met the recommendation of CDC, ACSM, and the surgeon general (4-5) for moderate-intensity physical activity (30 minutes five times per week) or vigorous physical activity (20 minutes three times per week) were excluded from the study and were not interviewed. (CDC does not include walking as one of the criteria for establishing physical activity status.) One month following the campaign (follow-up), the same respondents interviewed at baseline were contacted again by telephone in a panel design. TNS Intersearch Corporation (Westchester, Ill) conducted the surveys using a computer-assisted technology interview (CATI) system.

The telephone survey included 56 questions at baseline and 48 questions at follow-up. Baseline respondent telephone numbers were abandoned after 10 telephone calls at follow-up. Demographics, walking behavior, and moderate and vigorous physical activity were assessed with standard questions from CDC's Behavioral Risk Factor Surveillance System (BRFSS) (26). Questions from the Wheeling Walks intervention were used to address exposure and knowledge about campaign components (23,25). We asked questions about the source of media messages reported in Broome County only.

### Statistical analysis

The homogeneity of the demographic, health, and physical activity characteristics of the two communities was tabulated using bivariate contingency table chi-square tests. The initial outcome measures related to campaign exposure and recall. We established the change in walking behavior as a second outcome measure. We constructed dichotomous and continuous outcome variables. Dichotomous outcomes included the following: 1) change from nonactive walker to active walker from baseline to follow-up; 2) an increase in weekly walking time from baseline to follow-up; and 3) an increase of 30 minutes of weekly walking time from baseline to follow-up. An active walker was defined as an individual who walks at least 30 minutes per day 5 days per week. Continuous outcome measures, reported as change from baseline to follow-up, included the following: 1) number of days walked per week; 2) minutes walked per day; 3) minutes walked per week; and 4) total weekly minutes per week engaged in moderate or vigorous physical activity. Based upon previous studies (16,25,27), we stratified participants into four physical activity groups according to baseline activity level. Group 1 included participants who walked fewer than 10 minutes daily at baseline; Group 2 included participants who walked from 10 to 29 minutes daily at baseline; Group 3 included participants who walked from 30 to 60 minutes daily at baseline; and Group 4 included participants who walked more than 60 minutes daily at baseline.

Analyses were conducted using SAS software version 8.02 (SAS Institute Inc, Cary, NC). We compared the significance of dichotomous outcomes using chi-square tests and multiple logistic regression models to adjust for covariates. Continuous outcomes were compared between communities using the Wilcoxon rank sum test for median times and linear regression to adjust for covariates. The upper limit of weekly walk times was truncated to 840 minutes (28). Covariates were adjusted for logarithm-transformed body mass index (continuous), employed (binomial), fair to poor general health (binomial), and active walker at baseline (binomial).

## Results

### Process evaluation data

The media relations campaign activities resulted in 28 television news stories, 5 radio news stories, 10 newspaper stories, and 125 television news promotions. Pledges to walk daily were made by 10,800 individuals. The BC Walks Web site had 11,360 hits, and 961 individuals logged their minutes walked. The campaign speakers' bureau made 42 presentations that were attended by 1492 people. There were 30 worksite walking programs, which included 1207 employees and their family members who pledged to walk. Five schools, with approximately 2000 students, developed walking programs. In addition, the campaign distributed 250 prescription pads promoting the campaign to physicians and nurse practitioners in Broome County.

### Outcome evaluation data

Of the 1396 eligible respondents (aged 40 to 65) who were surveyed at baseline, 949 (68%) were identified as being insufficiently active. Of these 949 respondents, 575 resided in Broome County, and 374 resided in Chautauqua County. At follow-up, 393 (68%) of baseline respondents in Broome County and 207 (55%) of baseline respondents in Chautauqua County were reinterviewed. Table 2 shows the distribution of demographic and behavioral characteristics for participants with and without follow-up data. Most demographic characteristics were similar between intervention and control communities and between dropouts and completers (participants with follow-up information). However, compared with Chautauqua County, more Broome County completers reported fair to poor health and more reported being employed.

### Campaign awareness

In Broome County, the number of survey respondents who reported viewing any nonspecific media messages about walking or being more active increased from 61% at baseline to 81% at follow-up, compared with a decrease of 62% to 56% in Chautauqua County (*P* < .001 for the difference between the two counties) (data not shown). At baseline and follow-up, campaign recall was queried. Broome County survey respondents were asked if they had heard of BC Walks, and Chautauqua County respondents were asked if they had heard of Jamestown Walks, a fabricated name. Among Broome County respondents, 36% of respondents at baseline and 78% respondents at follow-up reported exposure to BC Walks; among Chautauqua County respondents, 19% of respondents at baseline and 17% of respondents at follow-up reported exposure to Jamestown Walks (*P* < .001 for the difference between the two counties).

Sixty-two percent of respondents reported exposure to television advertisements; 28% reported exposure to radio advertisements; 36% reported exposure to newspaper advertisements; 43% reported  exposure to television, radio, or newspaper news stories; 5% reported  exposure to worksite programs; and 4% reported exposure to educational programs and the speakers' bureau.

### Behavior change

Overall, there was a positive trend in the number of days spent walking in both the intervention and comparison community (Table 3). Although Group 1 participants in Broome County reported walking 1.8 days more per week at follow-up, and Group 1 participants in Chautauqua County reported walking 1.5 days more per week at follow-up, the 0.3-day difference between the two counties was not significant. Total minutes walked per week increased in Group 1 by 94 minutes and in Group 2 by 61 minutes in Broome County, compared with 70 minutes in Group 1 and 27 minutes in Group 2 in Chautauqua County. The 24-minute difference between Group 1 in each county and the 34-minute difference between Group 2 in each county, however, were not significant. Only the more sedentary Groups 1 and 2 showed gains in total minutes walked per week; declines were observed in the more active Groups 3 and 4. No significant differences or trends were observed for the nonwalking-related measures of moderate and vigorous activity.

Table 4 shows that 63% of Group 1 participants in Broome County showed any gain in minutes in weekly walking time, compared with 50% of Group 1 participants in Chautauqua County. In Broome County, 58% of Group 1 participants gained at least 30 minutes in weekly walking time, compared with 45% of Group 1 participants in Chautauqua County. Of all participants in Broome County, 47% reported any gain of weekly walking time, compared with 36% of all participants in Chautauqua County (adjusted odds ratio [OR], 1.66; 95% confidence interval [CI], 1.14–2.44). In addition, 41% of all participants in Broome County gained at least 30 minutes in weekly walking time, compared with 31% of all participants in Chautauqua County (adjusted OR, 1.56; 95% CI, 1.07–2.28). In Broome County, 16% of all participants changed from nonactive to active walkers, compared with 11% of all participants in Chautauqua County (adjusted OR, 1.71; 95% CI, 0.99–2.95).

## Discussion

There was a short-term impact of the BC Walks campaign in Broome County that was not seen in the comparison community. Process and impact data suggest that BC Walks faithfully replicated the Wheeling Walks intervention methodology in a larger community but that BC Walks achieved smaller effects.

Few studies have examined the repeatability of the additional walking questions asked in the BRFSS. In one Australian study using a representative population sample, the BRFSS walking question had acceptable repeatability, with an intraclass correlation of 0.45 (95% CI, 0.30–0.58). This was the second most reproducible of five survey-based walking estimates (29). Also, in this study, the additional walking questions on the BRFSS were more reproducible than the moderate- and vigorous-intensity physical activity questions in the BRFSS. Furthermore, the Australian estimate was also similar to one estimated for 106 American women, with an intraclass correlation for walking of 0.40 (95% CI, 0.23–0.55) (30). Moderate reliability and validity of the physical activity module of the BRFSS has been reported (31).

The increase in walking was smaller in Broome County than in Wheeling (23). No statistically significant difference was observed for categorical change from nonactive to active walker in the BC Walks campaign; the Wheeling Walks campaign resulted in a significant increase of 14% in the number of participants who changed from nonactive to active walker. Regression to the mean may explain the declines in walking behavior observed in the more active Groups 3 and 4.

Although data generally do not suggest weight management and reduction in obesity are associated with 30 minutes of moderate-intensity physical activity such as walking (5), the greater magnitude of behavior change for Wheeling Walks would suggest more postcampaign caloric expenditure. But even small populationwide changes in behavior have significant implications for reducing weight gain and the prevalence of obesity. In fact, the average American is gaining 0.5 to 1.0 kg per year (32). Increasing daily walking by approximately 5 minutes would account for 15 kcal, which would neutralize annual weight gain.

Preintervention to postintervention awareness of the BC Walks campaign among Broome County telephone respondents increased from 36% to 78%. No change of awareness was observed in the comparison community, indicating that the Broome County community was aware of the campaign message. BC Walks purchased the same amount of television and radio gross rating points as Wheeling Walks. Awareness of BC Walks was nearly as high as awareness of Wheeling Walks, with 78% of Broome County respondents reporting exposure to BC Walks and 90% of Wheeling respondents reporting exposure to Wheeling Walks.

BC Walks clearly created interest among media gatekeepers and generated 168 news stories and promotions on the campaign message during the 8 weeks of the campaign. During the first 8 weeks of Wheeling Walks, 280 television, radio, and print news stories were generated (25). A further comparison of campaign awareness based on type of media (Table 5) shows that although percentage of respondents reporting overall exposure was lower for BC Walks (78%) compared with Wheeling Walks (90%), the percentages are similar in both communities for reported exposure to television and radio. An exception is exposure to local news coverage: 81% of respondents in Wheeling indicated that they saw or heard news stories about the campaign; only 43% of the respondents in Broome County reported such exposure.

Several factors may have contributed to the diminished campaign impact of BC Walks compared with Wheeling Walks. Although the overall messages were the same in both campaigns (walk 30 minutes or more daily), a mass media telethon event held in Broome County in conjunction with the campaign kickoff to generate initial enthusiasm for walking encouraged callers to pledge to walk at least 10 minutes per day. The event was successful, with 10,800 community members making a pledge. The 10-minute message, however, may have created ambiguity. Although the 10-minute pledge was not inconsistent with the paid advertising campaign, which encouraged sedentary people to begin with 10 minutes, increase to 20 minutes, and then to walk 30 minutes daily, the 10,800 pledge respondents may have heard only the 10-minute daily message. Campaign implementation staff must ensure that all messages are designed and delivered to promote an established norm.

The fewer newspaper stories may also have lessened the impact of the campaign. Print news media may have a stronger impact on local community behavior than other media. The Broome County television market is more than double (890,890,573 viewers) that of Wheeling (418,170). Smaller media markets are easier to penetrate because they have fewer competing activities (20). In addition, the Wheeling Walks intervention staff spent 2 years planning implementation, which included a 12-week participatory process that increased social capital within the community (24). The participatory planning process resulted in the formation of community task forces to address barriers and resources associated with the development of Wheeling Walks. The Wheeling task forces were involved in conducting community focus groups as part of the formative research to develop campaign television, radio, and print advertisements. The participatory planning group developed into a community advisory commission (24). The mayor of Wheeling sanctioned the Walkable Wheeling Task Force, which he charged with providing semiannual progress reports on improvements to the walkability of the physical environment. The Wheeling community developed a great sense of ownership and engagement in the intervention. Although BC Walks had a communitywide commitment to increase physical activity and the regional metropolitan planning organization was actively involved, the process in Broome County was not as formalized at the local level as it was in Wheeling. (There was no participatory planning process, task force, community advisory commission, or environmental task force sanctioned by the mayor.) It is always a challenge to replicate programs and have the same efficacy achieved by the program originators.

Broome County was chosen as the intervention site because United Health Services Hospitals had established involvement with state and local stakeholders to promote physical activity in the region. Having only one intervention community and no random assignment limits the generalizability of the results. However, most of the large communitywide interventions reviewed for this study had similar methodologic limitations (8-11).

The BC Walks study, as in Welch Walks (a program in a West Virginia community even smaller than Wheeling [27]) and Wheeling Walks, demonstrated an increase in walking among participants (the purpose of the intervention), but it did not demonstrate an increase in moderate or vigorous activity. The positive change in walking behavior provides some evidence for the impact of targeted campaigns in smaller communities.

Future research should examine the potential effective mediators of change in social marketing campaigns. Additional trials should then empirically test the efficacy of interventions targeting the mediators. Public health intervention funding is limited and needs to be spent judiciously. Simultaneous or subsequent replication of small campaigns in similar communities will permit the empirical determination of relative cost-effectiveness of changing physical activity behavior and policy and environmental supports within communities.

## Figures and Tables

**Table 1:** Demographics of Intervention Community (Broome County, New York) and Comparison Community (Chautauqua County, New York), BC Walks, 2003

**Characteristic**	**Broome County**	**Chautauqua County**
Population, no.	200,536	139,750
Median age, y	38.2	37.9
Aged 40-65 y, no. (%)	63,298 (32)	44,030 (32)
Insufficiently active adults aged 40-65 y, no. (%)	36,080 (57)	25,097 (57)
Race		
African American, %	3.3	2.2
White, %	91.3	94.0
Per capita income, $	19,168	16,840
Unemployed, %	3.3	4.7
In labor force, %	60.5	61.4
Below poverty level, %	12.8	13.8

**Table 2:** Demographic and Behavioral Characteristics for Study Dropouts and Completers in Intervention Community (Broome County, New York) and Comparison Community (Chautauqua County, New York), BC Walks, 2003^a^

**Characteristic **	**Dropouts at Follow-up (n = 349) **		**Completers at Follow-up (n = 600) **	

	**Chautauqua Countyn = 167 **	**Broome Countyn = 182 **	**Chautauqua Countyn = 207 **	**Broome Countyn = 393 **
**Mean age, y (SD) **	52.0 (7.4)	52.6 (7.6)	52.2 (7.0)	52.4 (7.3)
**Female **	121 (72)	121 (66)	143 (69)	276 (70)
**White **	149 (89)	163 (90)	194 (94)	365 (93)
**Married or partnered **	94 (56)	115 (64)	134 (65)	256 (65)
**Education level **				
Some high school or less	18 (11)	8 (4)	13 (6)	31 (8)
High school or general equivalency degree	78 (48)	80 (45)	64 (31)	120 (31)
Some college or technical school	30 (18)	33 (18)	56 (27)	110 (28)
College graduate	38 (23)	58 (32)	74 (36)	132 (34)
**Employment status **				
Employed full time or part time or self-employed	105 (63)	120 (66)	116 (56)	255 (65)
Out of work or not in workforce	61 (37)	62 (34)	91 (44)	138 (35)^b^
**Income, $**				
<15,000	26 (19)	25 (16)	27 (14)	44 (13)
15,000-50,000	69 (50)	73 (47)	109 (56)	177 (51)
>50,000	44 (32)	56 (36)	57 (30)	127 (36)
**Fair to poor health **	71 (43)	79 (44)	76 (37)	189 (48)^c^
**Active walker, 30 min per day 5 days per week **	38 (23)	40 (22)	33 (16)	75 (19)
**Body mass index**				
Mean, kg/m2^2^ (SD)	29.2 (6.7)	28.4 (6.2)	28.9 (6.5)	28.9 (7.1)
<25.0, no. (%)	44 (29)	48 (29)	55 (28)	113 (31)
25.0-29.9, no. (%)	59 (38)	65 (40)	64 (33)	132 (36)
≥30.0, no. (%)	51 (33)	50 (31)	75 (39)	119 (33)

a All values are numbers with percentages in parentheses unless otherwise indicated. Not all participants responded to all questions.

b Difference between two counties determined by bivariate contingency table chi-square test; *P* = .03.

c Difference between two counties determined by bivariate contingency table chi-square test; *P* = .009.

**Table 3:** Change From Baseline Measures in Walking and Physical Activity Behavior Among Participants Who Completed Baseline and Follow-up Interviews in Intervention Community (Broome County, New York) and Comparison Community (Chautauqua County, New York), BC Walks, 2003

**County and Group^a^**	**No. of Participants in Group **	**Days Walked per Week, Mean(Median) **	**Min Walked per Day, Mean(Median) **	**Total Min Walked per Week, Mean(Median) **	**Total Min of Moderate to Vigorous Activity per Week, Mean(Median) **
**Chautauqua County **					
Group 1	73	1.5 (0)	17.3 (10)	69.9 (10)	57.0 (5)
Group 2	48	−0.8 (0)	5.4 (0)	27.1 (−5)	71.0 (30)
Group 3	63	−0.3 (0)	−12.1 (−15)	−15.1 (−40)	69.0 (30)
Group 4	20	−0.2 (0)	−54.5 (−60)	−401.5 (−10)	219.0 (135)
All respondents	204	0.2 (0)	−1.6 (0)	−12.6 (0)	77.8 (30)
**Broome County **					
Group 1	125	1.8 (2)	22.5 (15)	94.0 (45)	50.0 (0)
Group 2	125	−0.2 (0)	9.7 (0)	60.9 (20)	95.2 (25)
Group 3	98	0.1 (0)	−7.9 (−10)	−5.1 (−30)	79.2 (30)
Group 4	44	−0.5 (0)	−56.7 (−75)	−373.3 (−62)	149.4 (75)^b^
All respondents	392	0.5 (0)	1.9 (0)	6.3 (0)	82.2 (20)

a Participants were grouped according to baseline activity level. At baseline, Group 1 participants walked fewer than 10 minutes weekly; Group 2, from 10 to 29 minutes; Group 3, from 30 to 60 minutes; Group 4, more than 60 minutes.

b For this analysis, only 41 Group 4 respondents were available.

**Table 4:** Prevalence of Positive Change in Weekly Walking Time Among Participants Who Completed Baseline and Follow-up Interviews in Intervention Community (Broome County, New York) and Comparison Community (Chautauqua County, New York), BC Walks, 2003^a^

**County and Group^b^**	**No. of Participants in Group **	**Participants Who Gained Any Min, No. (%)**	** Participants Who Gained at Least 30 Min, No (%)**	** Participants Who Changed From Nonactive to Active Walker^c^, No. (%) **
**Chautauqua County **				
Group 1	73	37 (50)	33 (45)	5 (7)
Group 2	48	18 (38)	13 (27)	7 (15)
Group 3	63	18 (29)	17 (27)	7 (11)
Group 4	20	0 (0)	0 (0)	3 (15)
All respondents	204	73 (36)	63 (31)	22 (11)
All respondents, adjusted OR (95% CI)	204	Ref	Ref	Ref
**Broome County **				
Group 1	125	79 (63)	73 (58)	15 (12)
Group 2	125	72 (58)	54 (43)	28 (22)
Group 3	98	30 (31)	29 (30)	15 (15)
Group 4	44	4 (9)	4 (9)	5 (11)
All respondents	392	185 (47)	160 (41)	63 (16)
All respondents, adjusted OR (95% CI)	392	1.66 (1.14-2.44)	1.56 (1.07-2.28)	1.71 (0.99-2.95)

OR indicates odds ratio; CI, confidence interval; Ref, reference group.

a All values are numbers with percentages in parentheses unless otherwise indicated.

b Participants were grouped according to baseline activity level. At baseline, Group 1 participants walked fewer than 10 minutes weekly; Group 2, from 10 to 29 minutes; Group 3, from 30 to 60 minutes; Group 4, more than 60 minutes.

c Active walker is defined as an individual who walks at least 30 minutes per day 5 days per week.

**Table 5:** Comparison of the Wheeling Walks (Wheeling, WVa) and BC Walks (Broome County, New York) Campaigns to Promote Physical Activity

**Study Characteristic**	**Wheeling Walks**	**BC Walks**
Population, no.	31,420	200,536
Prime-time 30-s network television advertisements/gross rating points, no.	683/5104	953/4835
Drive-time 60-s radio advertisements/gross rating points, no.	1988/3461	1645/3245
30-s cable television advertisements, no.	1164	1314
Newspaper advertisements, no.	14	10
News stories, no.	173	43
Television news promotions, no.	107	125
Individuals who pledged to walk daily, no.	2248	10,800
Logged miles walked, no.	28,827	NA
Web site visits, no.	1530	11,360
Community presentations per attendees, no.	28/≥900	42/1492
Worksite presentations per employees, no.	40/3823	30/1207
Overall exposure, %	90	78
Individuals reporting they saw television advertisements, % surveyed postintervention	78	62
Individuals reporting they heard radio advertisements, % surveyed postintervention	34	28
Individuals reporting they saw or heard news stories, % surveyed postintervention	81	43
